# Burden of disease due to hip, knee, and unspecified osteoarthritis in the Peruvian social health insurance system (EsSalud), 2016

**DOI:** 10.12688/f1000research.22767.2

**Published:** 2020-08-17

**Authors:** Roger V. Araujo-Castillo, Carlos Culquichicón, Risof Solis Condor

**Affiliations:** 1 Dirección de Investigación, Instituto de Evaluación de Tecnologías en Salud e Investigación - IETSI, EsSalud, Lima, Peru; 2 CI-Emerge, Center of Emerging Diseases and Climate Change, Universidad Nacional de Piura, Piura, Peru; 3 Oficina de Inteligencia e Información Sanitaria, Gerencia Central de Prestaciones de Salud, EsSalud, Lima, Peru

**Keywords:** Osteoarthritis, burden of disease, disability adjusted life years, Peru, social inssurance

## Abstract

**Introduction: **Since its introduction by the World Health Organization (WHO), the concept of burden of disease has been evolving. The current method uses life expectancy projected to 2050 and does not consider age-weighting and time-discounting. Our aim is to estimate the burden of disease due to hip, knee, and unspecified osteoarthritis using this new method in the Peruvian Social Health Insurance System (EsSalud) during 2016.

**Methods: **We followed the original 1994 WHO study and the current 2015 Global Burden of Disease (GBD) methods to estimate disability adjusted life years (DALY) due to osteoarthritis, categorized by sex, age, osteoarthritis type, and geographical area. We used disability weights employed by the Peruvian Ministry of Health, and the last update issued by WHO.

**Results**: Overall, EsSalud reported 17.9 new cases of osteoarthritis per 1000 patients per year. Annual incidence was 23.7/1000 among women, and 72.6/1000 in people above 60 years old. Incidence was 5.6/1000 for knee osteoarthritis and 1.1/1000 for hip. According to the 1994 WHO method, there were 399,884 DALYs or 36.6 DALYs/1000 patients per year due to osteoarthritis. 12.4 and 2.2 DALYs/1000 patients per-year were estimated for knee and hip osteoarthritis, respectively. Using the 2015 GBD method, there were 1,037,865 DALYs or 94.9 DALYs/1000 patients per year. 31.4 and 5.3 DALYs/1000 patients per year were calculated for knee and hip osteoarthritis, respectively.

**Conclusions: **In the Peruvian social health insurance subsystem, hip, knee, and unspecified osteoarthritis produced a high burden of disease, especially among women and patients over 60. The 2015 GBD methodology yields values almost three times higher than the original recommendations.

## Introduction

Globally, the musculoskeletal disorders are a group of chronic diseases with high disability rates, which have been increasing, especially among people above 60 years old ^1, 2^. In Peru, osteoarthritis is the sixth cause of disability-adjusted life years (DALYs), representing 3% of global burden of disease ^3^. Knees and hip osteoarthritis produce the greatest disability in patients, with 6%, and 4% of the burden of disease due to knee and hip osteoarthritis, respectively ^1, 2^. Overall, the prevalence of osteoarthritis is homogeneous among men and women between 30 to 60 years old, but increases among women after that 4, 5.

The Peruvian burden of disease studies estimated that 308,804 DALYs were lost in the Peruvian population due to musculoskeletal disorders, representing 6% of the disease burden from all health conditions during 2004 ^6, 7^. Osteoarthritis was the health condition with the seventh greatest disability rates and caused 165,636 DALYs, which represents 3% of the total disease burden ^6, 7^. 98% of DALYs from osteoarthritis were attributed to the years lost due to disability (YLD), since osteoarthritis is not a primary cause of death. It mostly affected women, being the fourth biggest cause of DALYs in women ^6^. Among people between 45 and 59 years old, osteoarthritis was the second biggest cause of disability, with 109,804 DALYs, and in people above 60 years old represented 7% of total DALYs ^7– 9^. Without knowing the impact of osteoarthritis on people's lives, it is difficult to propose solutions, invest resources for prevention, and mitigate disability, especially among elders, who constitute a large group within the Peruvian social health insurance system (EsSalud), which cares for approximately 37% of people who seek medical attention ^10, 11^. Hence, there is an important need to estimate the burden of disease produced by osteoarthritis in this healthcare subsystem ^11^.

Since its introduction by the World Health Organization (WHO), the concept of burden of disease has been evolving. The original 1994 recommendations used life expectancy rates for men and women, gave less weight to the extremes of life, and penalized years when they were far from the current person age ^12^. The current method recommended by WHO in its 2015 Global Burden of Disease (GBD) study uses standard life expectancy are based on lowest observed mortality rates in any age group in populations over 5 million and does not time-discounting ^13^. Depending on the age distribution of a population, these new recommendations would probably yield higher estimates. Therefore, our aim is to estimate the burden of disease due to hip, knee, and unspecified osteoarthritis in EsSalud during 2016 using two different methods.

## Methods

### Population

Our study was conducted using nationwide data collected by the Peruvian social health insurance system during 2016. We included records of all patients older than 15 years who were attended due to osteoarthritis (International Classification of Diseases, ICD-10: M15-M19) between January and December 2016. We excluded patients whose ICD-10 codes for osteoarthritis were registered in hospitalization and patients with previous treatment.

All medical attentions at EsSalud between January and December 2016 were reviewed. These attentions had been registered using three different electronic systems. The Hospital Management System (SGH, by its acronym in Spanish) and the Health Services Management System (HSS, by its acronym in Spanish) record inpatient and outpatient attentions delivered at secondary and tertiary care level facilities. Meanwhile, the Health Information System for Primary Care Centers (SISCAP, by its acronym in Spanish) registers health attentions delivered at primary care facilities. All entries that fulfilled our selection criteria were included. In addition, we reviewed all death certificates issued by EsSalud during the study period, and selected those patients who had osteoarthritis registered as primary or contributory cause of death. Identities of patients on databases were kept confidential during data management and analysis.

### Variables

Variables included sex, age, health center, geographical area, osteoarthritis of the hip (ICD-10: M16), osteoarthritis of the knee (ICD-10: M17), osteoarthritis of first carpometacarpal joint (ICD-10: M18), polyosteoarthritis (ICD-10: M15), unspecified osteoarthritis (ICD-10: M19), death due to osteoarthritis, and time between initial attention for osteoarthritis and death. Additionally, the following indicators were estimated for each person:

*Life expectancy at time of care delivered:* Life expectancy was estimated according to two methods: The original Harvard burden of disease study from 1994 ^5^ employed the West extended model life tables from level 26 ^12^ to calculate life expectancy at the time the disability started (in our case, time of osteoarthritis diagnosis). It then applied an age-weighted function, defined by: Cxe ^-βx^, where “x” is the age in years, “C” is the constant of age weighting adjustment (value: 0.16458) and “β” is the age weighting parameter (value: 0.04). This function draws a curve assigning different weights to ages, giving greater values to adult ages since they were considered “more productive”. Additionally, a 3% discount by year was applied, trying to capture the fact that people appreciate years in the immediate future than those further away more. The second method, used in the 2015 GBD study, uses standard life expectancy are based on lowest observed mortality rates in any age group in populations over 5 million and does not time-discounting ^13^.
*Years of life lost (YLL):* In case the patient died because of osteoarthritis, the YLL was calculated as the life expectancy at the time of death using the two methods described above ^12, 13^. Living patients were assigned a zero value for YLL.
*Years lost due to disability (YLD):* YLDs were estimated as the average duration of the illness at age of onset, times the disability weight (0 = maximum health, 1 = death) ^12^. Given that osteoarthritis is a chronic condition that lasts until death, the average duration of the illness was considered as the life expectancy at the time of initial diagnosis. For this estimation, we used the two methods previously described ^12, 13^. Two disability weights were used: 0.165, which is the value employed by the 2015 GBD study for severe musculoskeletal diseases of lower limbs ^14, 15^; and 0.28, which was used in the Peruvian burden of disease studies for osteoarthritis severe enough to seek medical attention ^6– 10^.
*Disease-adjusted life years (DALY):* This was the sum of the years of life lost (YYL) and the years lost due to disability (YLD) for each patient.

### Statistical analysis

We described numerical variables using means and standard deviations. Categorical variables were described using frequencies and proportions. Osteoarthritis incidences were calculated by dividing the number of new cases registered by the number of insured patients in EsSalud during 2016. YLL, YLD, and DALYs were estimated, summing these metrics in total and by subgroups using the 1994 Harvard and the 2015 GDB methods, and the 2015 GBD and the Peruvian Ministry of Health (MINSA) disability coefficients, which means four iterations were calculated for each metric. In addition, DALYs incidence ratios per thousand patients per year were calculated by dividing total number of DALYs by the total number of insured patients registered in EsSalud during 2016. The statistical analysis used STATA v14.0 (Statacorp, College Station, Tx). Code used for the analysis is available as *Extended data* ^16^.

### Ethical statement

The Institutional Review Board (IRB) of the Edgardo Rebagliati Martins National Hospital (HNERM) approved this study (#832-2019-195). The IRB waived the requirement for consent from the patients as the study was conducted with an anonymized dataset.

## Results

During 2016, the Peruvian social health insurance system attended 196,003 patients for a first time visit due to osteoarthritis. Among them, 65.5% (n=128,323) were women, the mean age was 60.9±15.1 years, 62.6% (n=122,705) had polyosteoarthritis or unspecified osteoarthritis, 31.0% (n=60,788) had osteoarthritis of the knee, 5.9% (n=11,472) osteoarthritis of the hip and 0.5% osteoarthritis of other joints ^17^. We estimated 17.9 new cases of osteoarthritis per 1000 insured patients in 2016. The incidence of osteoarthritis in women was 23.7/1000 insured patients per year, and in patients over 60 years old the incidence was 72.6/1000 insured patients per year. The incidence of polyosteoarthritis or unspecified osteoarthritis was 11.2/1000 patients per year, the incidence of knee osteoarthritis was 5.6/1000 patients per year, and hip 1.1/1000 patients per year. The geographical region with higher incidence was the Northern Coast/Highlands with 20.2/1000 patients per year, and the lowest was found in the Amazon Rainforest with 13.3/1000 patients per year ( Table 1).

**Table 1.** Osteoarthritis incidence among patients from the Peruvian social health insurance system in 2016.

Parameter	New osteoarthritis cases	% col	Insured patients EsSalud 2016	Incidence/1000 insured patients
**Total**	196,003	100.0	10,937,079	17.9
**Gender**				
Male	67,680	34.5	5,521,152	12.3
Female	128,323	65.5	5,415,927	23.7
**Age**				
15–44	30,222	15.4	5,083,446	5.9
45–59	58,733	30.0	1,747,647	33.6
≥60	107,048	54.6	1,474,919	72.6
**Type of osteoarthritis**				
Polyosteoarthritis or unspecified	122,705	62.6	10,937,079	11.2
Knee osteoarthritis	60,788	31.0		5.6
Hip osteoarthritis	11,472	5.9		1.0
First carpometacarpal joint osteoarthritis	1,038	0.5		0.1
**Hospital service**				
Emergency	9,809	5.0	NA	NA
Outpatient	186,194	95.0	NA	NA
**Local health network**				
Metropolitan Lima	88,597	45.3	5,222,771	17.0
Northern Coast/Highlands	52,882	27.0	2,612,164	20.2
Southern Coast/Highlands	45,877	23.5	2,481,640	18.5
Amazon Rainforest	8,241	4.2	620,504	13.3

Since only three deaths certificates registered osteoarthritis as the primary cause of death, the DALYs corresponded mainly to the years lost due to disability (YLD). According to the 1994 Harvard methodology, osteoarthritis produced 399,884 DALYs using the latest WHO disability weights, or 678,591 DALYs if employing the Peruvian MINSA weights. Using these disability weights and after adjusting by the number of insured patients, osteoarthritis delivered 62.0 DALYs/1000 patients per year, poly/unspecified osteoarthritis 37.0 DALYs/1000 patients per year, knee osteoarthritis 21.0 DALYs/1000 patients per year, and hip osteoarthritis 3.8 DALYs/1000 patients per year ( Table 2). According to the 2015 GBD methodology, osteoarthritis produced 1,037,865 DALYs using the latest WHO disability weights, or 1,761,225 DALYs if employing the Peruvian MINSA weights. Using these disability weights and after adjusting by the number of insured patients, osteoarthritis delivered 161.0 DALYs/1000 patients per year, poly/unspecified osteoarthritis 97.2 DALYs/1000 patients per year, knee osteoarthritis 53.3 DALYs/1000 patients per year, and hip osteoarthritis 9.7 DALYs/1000 patients per year ( Table 2).

**Table 2.** DALYs due to osteoarthritis among patients from the Peruvian social health insurance system in 2016, using the 1994 Harvard and 2015 GBD methodologies.

Parameter	DALYs 1994 Harvard				DALYs 2015 GBD			
	Disability weight WHO (0.165)	DALYs /1000 insured patients	Disability weight MINSA (0.28)	DALYs /1000 insured patients	Disability weight WHO (0.165)	DALYs /1000 insured patients	Disability weight MINSA (0.28)	DALYs /1000 insured patients
**Total**	399,884	36.6	678,591	62.0	1,037,865	94.9	1,761,225	161.0
**Gender**								
Male	127,583	23.1	216,505	39.2	344,542	62.4	584,677	105.9
Female	272,301	50.3	462,086	85.3	693,323	128.0	1,176,548	217.2
**Age**								
15–44	128,200	25.2	217,551	42.8	277,680	54.6	471,214	92.7
45–59	154,492	88.4	262,168	150.0	383,677	219.5	651,088	372.6
≥60	117,193	79.5	198,872	134.8	376,508	255.3	638,923	433.2
**Type of osteoarthritis**								
Polyosteoarthritis or unspecified	238,170	21.8	404,167	37.0	626,203	57.3	1,062,648	97.2
Knee osteoarthritis	135,176	12.4	229,389	21.0	343,610	31.4	583,096	53.3
Hip osteoarthritis	24,473	2.2	41,530	3.8	62,653	5.7	106,320	9.7
First carpometacarpal joint osteoarthritis	2,066	0.2	3,505	0.3	5,399	0.5	9,161	0.8
**Local health network**								
Metropolitan Lima	170,001	32.5	288,486	55.2	448,276	85.8	760,711	145.7
Northern Coast/ Highlands	111,508	42.7	189,225	72.4	287,018	109.9	487,061	186.5
Southern Coast/ Highlands	98,099	39.5	166,471	67.1	251,533	101.4	426,844	172.0
Amazon Rainforest	19,492	31.4	33,078	53.3	48,973	78.9	83,106	133.9

DALY, disability-adjusted life year; WHO, World Health Organization; GBD, Global Burden of Disease; MINSA, Peruvian Ministry of Health

We analyzed the burden of disease by knee and hip osteoarthritis subgroups. For knee osteoarthritis, the incidence was 4.5 new cases/1000 patients per year among men and 6.7 new cases/1000 patients per year in women. People between 15–44 years old had only 2.4 new cases/1000 patients per year, whereas people aged 60 or over presented 20.2 new cases/1000 patients per year. According to the 1994 Harvard methodology and using the Peruvian MINSA disability weights, knee osteoarthritis produced 16.6 DALYs/1000 patient per year among men, and 25.5 DALYs/1000 patient per year in women. It was also responsible for 17.1 DALYs/1000 patient per year among people 15–44 years old, and 38.9 DALYs/1000 patient per year in patients over 60 years old ( Table 3).

**Table 3.** DALYs due to knee and hip osteoarthritis among patients from the Peruvian social health insurance system in 2016, using the 1994 Harvard and 2015 GBD methodologies.

	Incidence			DALYs 1994 Harvard				DALYs 2015 GBD			
	New cases	%col	Incidence/1000 insured patients	Disability weight WHO (0.165)	DALYs /1000 insured patients	Disability weight MINSA (0.28)	DALYs/1000 insured patients	Disability weight WHO (0.165)	DALYs /1000 insured patients	Disability weight MINSA (0.28)	DALYs/1000 insured patients
**Knee osteoarthritis**											
**Gender**											
Male	24,590	40.5	4.5	53,927	9.8	91,513	16.6	139,704	25.3	237,073	42.9
Female	36,198	59.6	6.7	81,248	15.0	137,876	25.5	203,906	37.6	346,023	63.9
**Age**											
15–44	12,023	19.8	2.4	51,205	10.1	86,893	17.1	110,923	21.8	188,233	37.0
45–59	19,040	31.3	10.9	50,155	28.7	85,111	48.7	124,769	71.4	211,730	121.2
≥60	29,725	48.9	20.2	33,816	22.9	57,385	38.9	107,918	73.2	183,134	124.2
**Hip osteoarthritis**											
**Gender**											
Male	3,867	33.7	0.7	7,623	1.4	12,936	2.3	20,342	3.7	34,519	6.3
Female	7,605	66.3	1.4	16,850	3.1	28,594	5.3	42,311	7.8	71,801	13.3
**Age**											
15–44	2,086	18.2	0.4	9,054	1.8	15,365	3.0	19,506	3.8	33,102	6.5
45–59	3,347	29.2	1.9	8,779	5.0	14,897	8.5	21,826	12.5	37,038	21.2
≥60	6,039	52.6	4.1	6,640	4.5	11,268	7.6	21,321	14.5	36,181	24.5

DALY, disability-adjusted life year; WHO, World Health Organization; GBD, Global Burden of Disease; MINSA, Peruvian Ministry of Health.

Regarding hip osteoarthritis, the incidence was 0.7 new cases/1000 patients per year among men and 1.4 new cases/1000 patients per year in women. People between 15–44 years old had only 0.4 new cases/1000 patients per year, whereas people aged 60 or more presented 4.1 new cases/1000 patients per year. According to the 1994 Harvard methodology and using the Peruvian MINSA disability weights, knee osteoarthritis produced 2.3 DALYs/1000 patients per year among men, and 5.3 DALYs/1000 patients per year in women. It was also responsible for 3.0 DALYs/1000 patients per year among people 15–44 years old, and 7.6 DALYs/1000 patient per year in patients over 60 years old ( Table 3).

## Discussion

The incidence for osteoarthritis overall was 17.9 new cases/1000 insured patients per year, for poly/unspecified osteoarthritis was 11.2/1000 patients per year, for knee osteoarthritis was 5.6/1000 patients per year, and for hip osteoarthritis was 1.0/1000 patients per year. These findings are consistent with estimations previously made in Peru and high-income countries. In Peru during 2009, the incidence of knee osteoarthritis was 3.26/1000 inhabitants per year, and for hip osteoarthritis was 0.91/1000 inhabitants per year ^8^. In Canada, the incidence of all osteoarthritis was 8.6 new cases/1000 inhabitants per year with a prevalence of 80.3 cases/1000 inhabitants until 2015 ^18^. In Spain there was an incidence of 6.5 new cases/1000 inhabitants per year for knee osteoarthritis and 2.1/1000 inhabitants per year for hip osteoarthritis during 2014 ^19^. The findings of the 2010 GBD Study estimated a prevalence of 38 cases/1000 inhabitants per year for knee osteoarthritis and 8.5 cases/1000 inhabitants per year for hip osteoarthritis ^20^.

Considering MINSA had used the 1994 Harvard methodology and their own disability weights in their previous burden of disease studies, we will follow suit in order to compare our results with previous reports. Hence, the estimated burden for overall osteoarthritis was 62.0 DALYs/1000 patients per year, for poly/unspecified osteoarthritis was 37 DALYs/1000 patients per year, for knee osteoarthritis was 21 DALYs/1000 patients per year, and for hip osteoarthritis was 3.8 DALYs/1000 insured patients per year ^21^. These estimates are consistently higher than the disease burden of osteoarthritis reported by all studies conducted in Peru under the 1994 Harvard methodology. Since 2006, the General Directorate of Epidemiology (DGE, by its acronym in Spanish) of MINSA, which has carried out studies to estimate the osteoarthritis disease burden in Peruvian settings, initially estimated 94,160 DALYs due to osteoarthritis (3.4 DALYs/1000 inhabitants) ^3^. For 2009, the DGE estimated 165,636 DALYs due to osteoarthritis ^7^. In 2012, osteoarthritis delivered 193,774 DALYs (6.4 DALYs/1000 inhabitants) ^9^. In 2015, EsSalud carried out a study of the burden of disease among the insured patients and estimated 131,220 DALYs due to osteoarthritis (12.3 DALYs/1000 insured) ^10^.

As observed in Table 4, there has an increment in DALYs despite using the same methodology. This could be due to different approaches to measure incidence. Previous studies have used a variety of sources including epidemiological surveillance, number of attentions, population-based surveys, medical chart reviewing, and scientific papers ^6– 10^. It is possible then, that the increment is just echoing a better registry of cases. In the present study, we only used electronic records of attentions, and employed all national data instead of small samples. This could lead to better capturing of cases, otherwise overlooked in previous studies. On the other hand, it is possible that our data collection strategy overestimates the number of new cases. Since we based our estimations on the ICD-10 diagnosis entered for each attention, it is not certain that all cases were confirmed at the time of the medical visit. In addition, we could have included mild cases not usually considered for surveillance or research purposes.

**Table 4.** Comparison of DALYs due to osteoarthritis between different studies in Peru 2016, using the 1994 Harvard methodology and the MINSA disability weight.

	MINSA 2006	MINSA 2008	MINSA 2009	MINSA 2012	EsSalud 2014	EsSalud 2016 (Using only ICD-10 for specific site osteoarthritis)	EsSalud 2016 (Using All ICD-10 codes for osteoarthritis)
**Total**	94,160	97,115	165,636	193,774	131,220	274,424	678,591
Per thousand patients	3.4	3.8	-----	6.4	12.3	25.1	62.0
**Males**	42,664	39,830	65,864	79,550	43,140	105,476	216,505
Per thousand patients	3.1	3.2	-----	5.3	8.0	19.1	39.2
**Females**	51,496	57,285	99,773	114,225	88,079	168,948	462,086
Per thousand patients	3.8	4.3	-----	7.6	16.7	31.2	85.3
**15–44 years**	-----	-----	-----	45,638	32,694	103,262	217,551
Per thousand patients	-----	-----	-----	3.0	6.7	20.3	42.8
**45–59 years**	-----	-----	-----	75,924	50,534	101,433	262,168
Per thousand patients	-----	-----	-----	18.7	30.8	58.0	150.0
**≥60 years**	-----	-----	-----	72,212	47,991	69,729	198,872
Per thousand patients	-----	-----	-----	26.0	34.2	47.3	134.8

DALY, disability-adjusted life year; WHO, World Health Organization; MINSA, Peruvian Ministry of Health; EsSalud: Peruvian social health insurance system; ICD, International Classification of Diseases.

We also observed discrepancies when stratified by age group. In our study, the estimated DALYs were higher for insured patients between 45–59 years old, with 150.0 DALYs/1000 patients per year, followed by people older than 59 years, with 134.8 DALYs/1000 patients per year, and people between 15–44 years old, with 42.8 DALYs/1000 patients per year. In contrast, the 2012 Peruvian study found that the elderly group produced the most DALYs/1000 people ^9^. The same study found that the elderly group delivered a burden of disease almost nine times the one registered for 15–44 years old people (26.0 vs 3.0 DALYS/1000 inhabitants); meanwhile, our study found a much more reduced gap of only three times (134.8 vs 42.8 DALYS/1000 inhabitants). One possibility is that our study identified more patients with osteoarthritis in the younger group than previous studies.

Estimations of burden of disease would differ depending on the methods used and the disability coefficients assigned to the disease. The use of different life expectancy values (West 26 vs GBD 2050) and weights/discounts affects not only the absolute values of DALYs but also the estimations within subgroups ^13^. In addition, the original 1994 Harvard method differentiated life expectancy values for men (80 years) and women (82.5 years), penalized the extremes of life ages, and discounted the value of years away in time, reducing the DALYs contributed by men and younger people ^22– 24^. The 2015 GBD method tried to correct these differences by using the maximum projected life expectancy for 2050 (91.9 years) without differences between sexes, and discarded age-weighting and time-discounts. The intended effect is to increase the sensitivity of the method to estimate DALYs, especially in the extremes of life. In our study, the 2015 GBD methodology yielded values almost three times higher than the original recommendations, and reduced the gaps between sexes and age groups.

Another important component when calculating DALYs is the disability weights. The original 1994 Harvard methodology recommends using a disability weight of 0.22 for diseases that limit recreation, occupation, education or procreation activities, and 0.40 if a disease limits two or more of these activities. Instead, the 2015 GBD methodology recommends using different weights depending on the severity and location of the disease, giving a disability factor for hip and knee osteoarthritis of 0.165, corresponding to musculoskeletal problems, lower limbs, severe. On the other hand, the MINSA and EsSalud studies have consistently used a 0.28 weight for osteoarthritis regardless of age or sex, because it considers this disease severe enough to seek medical attention. Using the MINSA disability weights instead of the 2015 GBD recommendations increases the absolute values of DALYs by approximately 70%.

## Conclusions

In the Peruvian social health insurance subsystem, which covers almost 40% of the population, polyosteoarthritis, unspecified osteoarthritis, knee and hip osteoarthritis produced a high burden of DALYs lost, especially among patients over 60 years old and women. The 2015 GBD methodology yields values almost three times higher than the original recommendations, and the disability weights used by MINSA produced estimates 70% higher than using the 2015 GBD weights.

## Data availability

### Underlying data

Figshare: db_osteoarthritis_v3.xlsb http://doi.org/10.6084/m9.figshare.12003957 ^17^

Data are available under the terms of the Creative Commons Zero "No rights reserved" data waiver (CC0 1.0 Public domain dedication).

### Extended data

Zenodo: culquichicon/gbd-ostheoarthritis: Global burden of disease in osteoarthritis in Peru https://doi.org/10.5281/zenodo.3727167 ^16^

Data are available under the terms of the GNU General Public License version 3 (GPL-3.0).
