# Supplementary material for: Burden of disease due to hip, knee, and unspecified osteoarthritis in the Peruvian social health insurance system (EsSalud), 2016
Source: F1000Res. 2020 Aug 17;9:238. Originally published 2020 Apr 3. [Version 2] doi: 10.12688/f1000research.22767.2 (PMC7443783; doi:10.12688/f1000research.22767.2)
Supplement: Peer review history

# Peer review: Reviewer response for version 2

**DOI:** 10.5256/f1000research.28733.r69449

**Published:** 2020-08-24

**Authors:** Mario Valladares-Garrido

**Affiliations:** 1 Universidad Continental, Lima, Peru

It seems the authors properly implemented Dr. Vos' suggestions across the introduction, and methods section, besides Dr. Pereyra's suggestion on the discussion. Results seem to be valid considering Peru is still using previous versions of GBD methods to report its metrics and make a good reflection on a chronic condition in elderly population of Peru.

Is the work clearly and accurately presented and does it cite the current literature?

Yes

If applicable, is the statistical analysis and its interpretation appropriate?

Yes

Are all the source data underlying the results available to ensure full reproducibility?

Yes

Is the study design appropriate and is the work technically sound?

Yes

Are the conclusions drawn adequately supported by the results?

Yes

Are sufficient details of methods and analysis provided to allow replication by others?

Yes

Reviewer Expertise:

Global health, internal medicine, mental health.

I confirm that I have read this submission and believe that I have an appropriate level of expertise to confirm that it is of an acceptable scientific standard.

# Peer review: Reviewer response for version 2

**DOI:** 10.5256/f1000research.28733.r69450

**Published:** 2020-08-20

**Authors:** Reneé Pereyra-Elías

**Affiliations:** 1 University of Oxford, Oxford, UK

I think the authors have addressed the observations I raised. However, as I mentioned, I am not an expert in the construction of the "Burden of Disease" metrics. It would be important to have the opinion of Prof. Theo Vos on the revised version of the manuscript. If these matters have been appropriately addressed, I think the manuscript can be indexed.

Is the work clearly and accurately presented and does it cite the current literature?

Yes

If applicable, is the statistical analysis and its interpretation appropriate?

Yes

Are all the source data underlying the results available to ensure full reproducibility?

Yes

Is the study design appropriate and is the work technically sound?

Partly

Are the conclusions drawn adequately supported by the results?

Yes

Are sufficient details of methods and analysis provided to allow replication by others?

Yes

Reviewer Expertise:

Social epidemiology, mental health, medical education.

I confirm that I have read this submission and believe that I have an appropriate level of expertise to confirm that it is of an acceptable scientific standard.

# Peer review: Reviewer response for version 1

**DOI:** 10.5256/f1000research.25137.r64466

**Published:** 2020-06-22

**Authors:** Reneé Pereyra-Elías

**Affiliations:** 1 University of Oxford, Oxford, UK

This manuscript covers an important topic, that has not been explored much in Peru. Due to its relevance and the population included in the study (all those insured by EsSalud, that provides insurance to a significant fraction -about 1/3- of the Peruvian population), I consider it is worth publishing. However, it is necessary to review some important points. 

- I think the main aspect to review would be the construction of the 'burden of disease' variables. I am not an expert in these metrics. I highly recommend reading and considering the detailed suggestions of reviewer Prof. Theo Vos, who is a world expert in the field. 

**Minor revisions:**

- Sample size: The authors state that they were trying to detect differences between subgroups? Which subgroups? Knee and hip osteoarthritis? Males and females? Age groups? There are no hypothesis tests reported throughout the manuscript. Please clarify that. 
- It would be interesting to see some potential explanations for the regional differences between the burden of disease. Is it due to the timing of diagnosis? Is it due to the characteristics of these populations?
- The manuscript might benefit from an English-language edit.

Is the work clearly and accurately presented and does it cite the current literature?

Yes

If applicable, is the statistical analysis and its interpretation appropriate?

Yes

Are all the source data underlying the results available to ensure full reproducibility?

Yes

Is the study design appropriate and is the work technically sound?

Partly

Are the conclusions drawn adequately supported by the results?

Yes

Are sufficient details of methods and analysis provided to allow replication by others?

Yes

Reviewer Expertise:

Social epidemiology, mental health, medical education.

I confirm that I have read this submission and believe that I have an appropriate level of expertise to confirm that it is of an acceptable scientific standard, however I have significant reservations, as outlined above.

# Peer review: Reviewer response for version 1

**DOI:** 10.5256/f1000research.25137.r62049

**Published:** 2020-06-04

**Authors:** Mario Valladares-Garrido

**Affiliations:** 1 Universidad Continental, Lima, Peru

Overall, the findings match with Peruvian estimates of the global burden of disease (GBD) in osteoarthritis done by the Peruvian Ministry of Health and the Social Health Insurance in their previous reports over the years.

 A study strength is that the authors report both methodologies (1994 and 2015) for the global burden of disease estimations, in that sense, they address a gap of knowledge between Peruvian reports.

 This study contributes to Peruvian GBD estimations especially in chronic non-communicable diseases with great impacts on elderly people.

Is the work clearly and accurately presented and does it cite the current literature?

Yes

If applicable, is the statistical analysis and its interpretation appropriate?

Yes

Are all the source data underlying the results available to ensure full reproducibility?

Yes

Is the study design appropriate and is the work technically sound?

Yes

Are the conclusions drawn adequately supported by the results?

Yes

Are sufficient details of methods and analysis provided to allow replication by others?

Yes

Reviewer Expertise:

Global health, internal medicine, mental health.

I confirm that I have read this submission and believe that I have an appropriate level of expertise to confirm that it is of an acceptable scientific standard.

# Peer review: Reviewer response for version 1

**DOI:** 10.5256/f1000research.25137.r62054

**Published:** 2020-04-20

**Authors:** Theo Vos

**Affiliations:** 1 Institute for Health Metrics and Evaluation, Department of Health Metrics Sciences, School of Medicine, University of Washington, Seattle, WA, USA

This paper reports on a burden of disease analysis of osteoarthritis for Peru. Unfortunately, the researchers do not display adequate knowledge of current or past methods of burden of disease measurement. What they have labelled as their GBD2015 methods is very different from the actual GBD 2015 methods. The prior method is wrongly labelled as WHO method. In the 1990s WHO was not a formal partner with Harvard University and World Bank in producing the earliest round of GBD.

 I highlight the main errors in the introduction and methods sections below. As these variations to established methods make comparisons impossible, I refrain from making further comments on the results and discussion.

  

- Page 3, second sentence: a global estimate of OA DALYs is referenced to a Peruvian study.
- Page 3, third paragraph:
  Unclear statement ‘…tables based on 1966 data…’.
  “…employed different life expectancy for men and women…”; what is meant is that the standard life expectancy against which years of life lost are estimated had a difference between men and women.
  “..2015 Global Burden of Disease (GBD) study ..”; this is now three iterations of GBD out-of-date …though these general methods still apply.
  “..life expectancy projected to 2050 ..; not correct. I presume it refers to new approach to standard life expectancy which is same for men and women and is based on lowest observed mortality rates in any age group in populations over 5 million.
- Page 3, last paragraph: “…We excluded patients whose ICD-10 codes for osteoarthritis were registered.
- "during hospitalization and patients who were previously treated for osteoarthritis….”; how?
- Page 3/4: “…Sample size calculation estimated that a minimum of 20,000 records were needed to find differences of at least four DALYs/1000 people between subgroups with 80% power and 95% significance.”; no reason to sample when using medical claims data.
- Page 4, second paragraph: “Covariates…”, better to use term ‘Variables…’.
- Page 4: ‘The original WHO burden of disease study from 1994 ..’; This was a study by Harvard University for World Bank, not WHO.
- Page 4: ‘The second method, employed in the 2015 GBD study, uses the maximum worldwide life expectancy projected to 2050…’ Incorrect statement; see comment above.
- Page 4: ‘…the YLL was calculated as the life expectancy at the time of death using the two methods described above12,13…’; the two references are both for methods prior to GBD 2010.
- Page 4: ‘..Given that osteoarthritis is a chronic condition that lasts until death, the average duration of the illness was considered as the life expectancy at the time of initial diagnosis. For this estimation, we used the two methods previously described ^12,13^…’; same comment as previous, that the two references refer to methods prior to GBD2010 only. What is not stated is a big methods change in non-fatal estimation from GBD2010 onwards estimating YLDs based on prevalence and not incidence. Thus, the authors incorrectly describe GBD2015 methods and their results, therefore, are not comparable to those of GBD 2015 (or any iteration after GBD 2010).
- Page 5: ‘……Two disability weights were used: 0.165, which is the value employed by the 2015 GBD study for severe musculoskeletal diseases of lower limbs ^14,15^..’; question 1: why this selective choice of applying the more severe of DWs to all cases?; question 2: where did the Peruvian DW come from? What method was used?

Is the work clearly and accurately presented and does it cite the current literature?

No

If applicable, is the statistical analysis and its interpretation appropriate?

No

Are all the source data underlying the results available to ensure full reproducibility?

No

Is the study design appropriate and is the work technically sound?

No

Are the conclusions drawn adequately supported by the results?

No

Are sufficient details of methods and analysis provided to allow replication by others?

No

Reviewer Expertise:

NA

I confirm that I have read this submission and believe that I have an appropriate level of expertise to state that I do not consider it to be of an acceptable scientific standard, for reasons outlined above.
